# The chemistry of stalked barnacle adhesive (*Lepas anatifera*)

**DOI:** 10.1098/rsfs.2014.0062

**Published:** 2015-02-06

**Authors:** Jaimie-Leigh Jonker, Liam Morrison, Edward P. Lynch, Ingo Grunwald, Janek von Byern, Anne Marie Power

**Affiliations:** 1 School of Natural Sciences, National University of Ireland, Galway, Republic of Ireland; 2 Department of Mineral Resources, Geological Survey of Sweden, 75128 Uppsala, Sweden; 3 Department Adhesive Bonding and Surfaces, Fraunhofer Institute for Manufacturing Technology and Advanced Materials (IFAM), Group BioInspired Materials, 28359 Bremen, Germany; 4 Ludwig Boltzmann Institute for Experimental and Clinical Traumatology, Austrian Cluster for Tissue Regeneration, Donaueschingenstrasse 13, 1200 Vienna, Austria

**Keywords:** bioadhesive, cross-linking, barnacle, protein, spectroscopy

## Abstract

The results of the first chemical analysis of the adhesive of *Lepas anatifera*, a stalked barnacle, are presented. A variety of elements were identified in scanning electron microscopy with energy dispersive spectrometry (SEM-EDS) of the adhesive, including Na, Mg, Ca, Cl, S, Al, Si, K and Fe; however, protein–metal interactions were not detected in Raman spectra of the adhesive. Elemental signatures from SEM-EDS of *L. anatifera* adhesive glands were less varied. Phosphorous was mostly absent in adhesive samples; supporting previous studies showing that phosphoserines do not play a significant role in adult barnacle adhesion. Disulfide bridges arising from Cys dimers were also investigated; Raman analysis showed weak evidence for S–S bonds in *L. anatifera*. In addition, there was no calcium carbonate signal in the attenuated total reflectance Fourier transform infrared spectra of *L. anatifera* adhesive, unlike several previous studies in other barnacle species. Significant differences were observed between the Raman spectra of *L. anatifera* and *Balanus crenatus*; these and a range of Raman peaks in the *L. anatifera* adhesive are discussed. Polysaccharide was detected in *L. anatifera* adhesive but the significance of this awaits further experiments. The results demonstrate some of the diversity within barnacle species in the chemistry of their adhesives.

## 1. Introduction

Barnacles are the only sessile crustaceans; they glue the base of their bodies to a variety of materials, both natural [1] and man-made [2]. They achieve this over a range of submersion regimes, from intertidal semi-terrestrial conditions to fully submerged oceanic contexts. Uncovering the molecular mechanisms of adhesion in this system may be used in the prevention of biofouling and for various biomimetic applications, as has happened in mussels [3,4]. However, despite years of dedicated study, it is still not clear which molecular mechanisms allow barnacles to permanently adhere to surfaces underwater. Recent investigative approaches to answer this question have included ultrastructure of the adhesive system, characterization of adhesive proteins, predictions of their secondary structure, examinations of the bulk adhesive properties on various materials, settlement assays on functionalized surfaces and the mechanical properties of the adhesive [5–11]. Evidence is accumulating that the barnacle adhesive system does not use either of the more common molecular adhesive mechanisms: phosphorylated serines (PSer) or L-3,4-dihydroxyphenylalanine (DOPA) [5,12], which raises the possibility of finding adhesive novelties in the barnacle model.

Although Raman spectroscopy has been applied once before to acorn barnacle adhesive [13], this is only the second comprehensive chemical analysis (i.e. scanning electron microscopy with energy dispersive spectrometry (SEM-EDS), Raman and Fourier transform infrared (FTIR) spectroscopy) of the adhesive in a species of stalked barnacle [14]. These analyses, combined with structural investigations, could provide a deeper understanding of the barnacle adhesive, as they have done for caddisfly [15], tubeworm [16] and mollusc adhesives [17]. For example, high P content in the tubeworm adhesive indicated the non-standard amino acid PSer in that model [16]. Similarly, transition metals (including Zn, Fe, Cu and Mn) observed in mollusc adhesive gels were associated with important solubility and stiffening effects on these gels, possibly via catalysis or coordination of cross-linking [17]. Elemental investigations and FTIR spectroscopy have highlighted Cu–DOPA [18] and Fe–DOPA complexes, which are thought to have an important role in mussel adhesion [19–21]. Furthermore, adhesive complexes with Ca and Mg ions have been found in tubeworm, which potentially have a functional role via divalent interactions with phosphoprotein [16]. However, information for metal/inorganic involvement in the barnacle adhesive mechanism is generally lacking and there have only been two elemental characterizations using SEM-EDS, with both of these studies taking place in the balanomorph acorn barnacles [8,22]. Previous FTIR studies of barnacle adhesive using deconvolution methods have revealed the presence of amyloid-like β-sheet structures and phenolic signatures [23,24].

The benefits of SEM-EDS compared to methods which are more sensitive or quantitatively precise is that the elements can be mapped *in situ* within the gland or adhesive interface. The FTIR spectra of proteins are dominated by amide bands arising from the primary structure (protein backbone) and are not generally used to deduce specific amino acid side chain vibrations, although they are sensitive to sulfated and phosphorylated forms of biological molecules and phenolic groups [25]. By contrast, Raman spectra of proteins contain peaks characteristic of amino acid side chains [26–28] and the side chains of aromatic amino acids in particular give intensive Raman peaks [29]. However, for adult barnacle adhesive, Raman spectroscopy has thus far been used only once [13]. In addition to this, a single Raman study exists for barnacle larval adhesive [30].

The current study aims to use the above elemental mapping and spectroscopic techniques to investigate the adhesion of the stalked barnacle *Lepas anatifera*. This will add information about a taxon of barnacles with contrasting characteristics to the acorn barnacle species which have dominated previous investigations. These stalked barnacles (order Lepadiformes) have a very different morphology to acorn barnacles (electronic supplementary material, figure S1); they are larger bodied and their adhesive plaque is formed at the terminus of the relatively soft, flexible stalk or ‘peduncle’. More significantly, they possess a membrane at the base of the stalk in place of a calcareous baseplate, the latter being the norm in the species of balanomorph acorn barnacles that have dominated previous barnacle studies. Unlike their calcareous-based counterparts, no interference occurs from the base or lateral shell plates in *L. anatifera*. By adding information across the taxonomic breadth of barnacles, we hope to contribute information which distinguishes between features that are highly conserved for barnacle adhesion, more generally, and those which are adaptive for particular species or habitats. A recent study has shown that several homologous adhesive proteins are shared between acorn and stalked barnacles in different taxonomic orders [31], however, adhesive gene and amino acid sequence similarity can be rather low across these groups.

## 2. Material and methods

### 2.1. Sample collection

**Figure 1.**
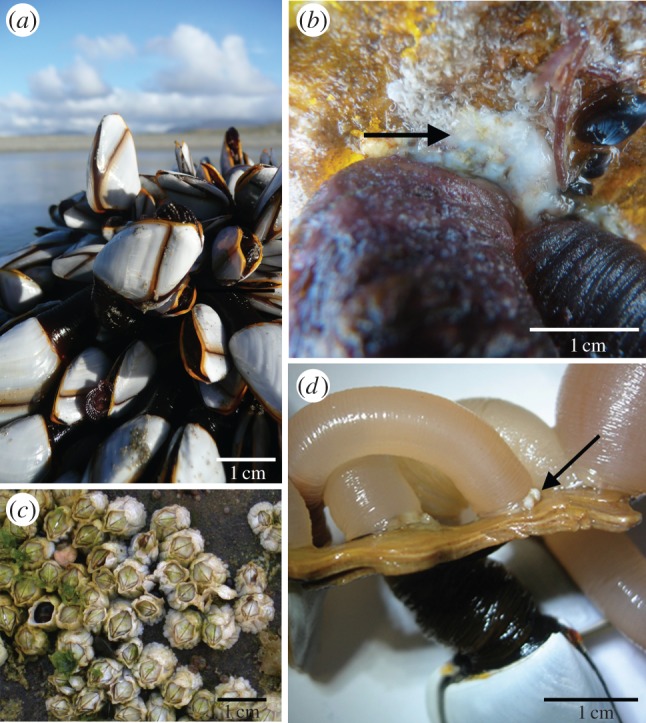
(*a*) *Lepas anatifera* stranded at the high-tide mark in county Mayo, Ireland. (*b*) *Lepas anatifera* adhered to a metal buoy (despite presence of yellow anti-fouling paint), with the opaque white rubbery adhesive visible adjacent to the pair of individuals (adhesive is indicated by an arrow); (*c*) Acorn barnacles (*Balanus* sp.) attached to intertidal rocks (note difference in scale). (*d*) Adhesive of *L. anatifera* on wood, with adhesive indicated by an arrow. (Online version in colour.)

Samples of live *L. anatifera* were collected opportunistically from the wild, having been washed up after storms on beaches in counties Galway, Mayo and Clare in the west of Ireland. Substrates included painted metal (data buoys), glass, plastic nylon rope and wood. *Lepas anatifera* was observed to produce a thick, opaque adhesive plaque on these substrates (figure 1). The adhesive generally had a rubbery consistency and small pieces could easily be pulled away from the cuticle of the barnacle with sterile forceps. Where possible, large pieces of adhesive were carefully removed from inside the adhesive plaque, which is located at the base of the peduncle. This was carried out using a clean razor blade and the surfaces that had been exposed to the outside environment were cut away. Samples were washed in ddH_2_O, and clean adhesive was then placed directly into Eppendorf tubes, stored at −70°C and subsequently freeze-dried at −50°C (Labconco Stoppering Tray Dryer) and stored in a desiccator.

### 2.2. Scanning electron microscopy with energy dispersive spectrometry

**Figure 2.**
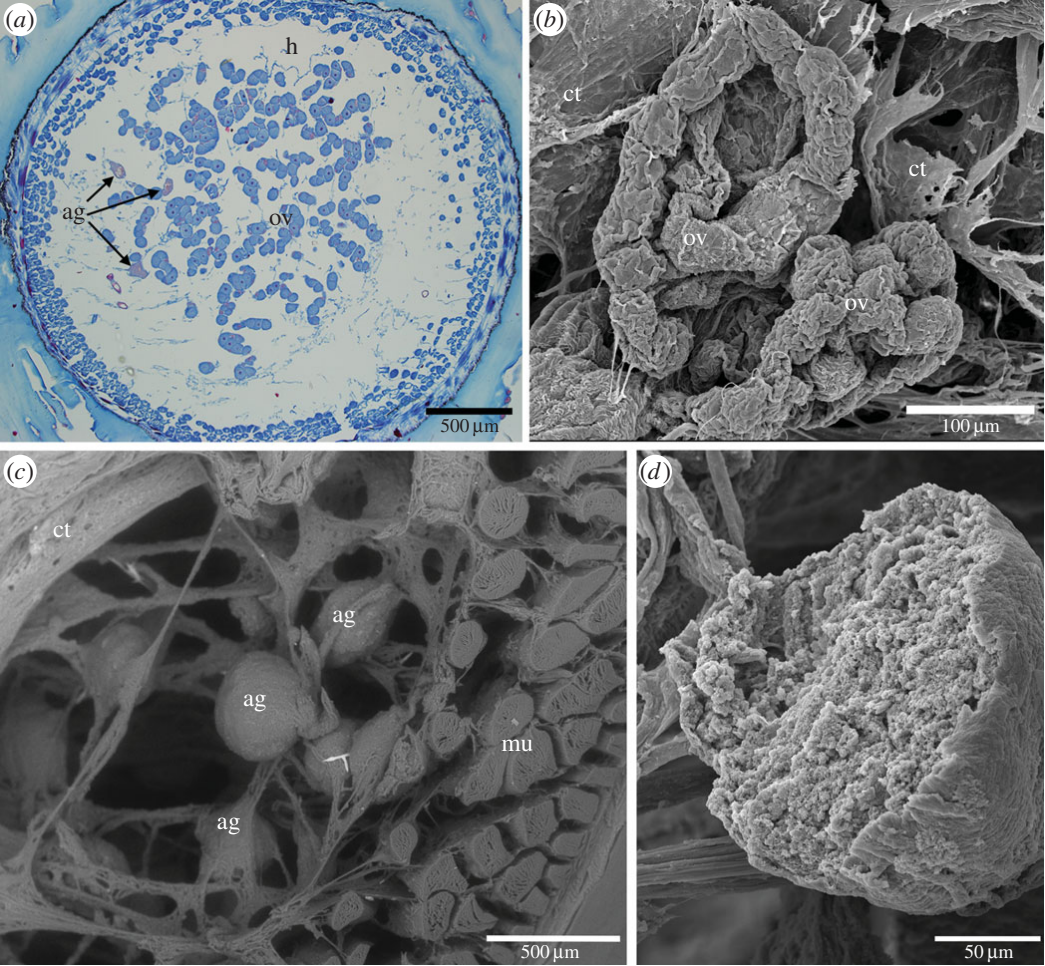
(*a*) Light microscope image of transverse section through *L. anatifera* peduncle, stained with Azan; (*b*) SEM image of the ovarian tubules and connective tissue; (*c*) SEM image of the adhesive glands, muscle and connective tissue; (*d*) high magnification view of a ruptured adhesive gland cell, showing a relatively smooth surface and tightly packed globular contents. Ag, adhesive glands; ct, connective tissue; h, haemolymph vessel; mu, muscle; ov, ovarian tissue. (Online version in colour.)

Four specimens of *L. anatifera* were fixed in Carnoy's fixative [32] and dehydrated in 70% alcohol before being sectioned by hand using a sharp razor blade. Prior to SEM, the sections were first viewed under a stereomicroscope (Olympus SZX16) while still immersed in ethanol. They were subsequently viewed in SEM environmental backscatter mode. The transition from light microscopy to electron microscopy allowed the various structures to be easily located and identified, including adhesive gland cells (figure 2). Freeze-dried samples of gland tissues were gold-coated for SEM, whereas freeze-dried adhesive from six different specimens of *L. anatifera* was carbon-coated for SEM. Investigations of the fine structure and chemistry of the adhesive glands and adhesive were performed using SEM in backscatter and secondary emission mode (Hitachi S-4700, acceleration voltage 20 kV, emission current 10 µA, working distance 12 mm). Spatial elemental distribution mapping using energy dispersive spectrometry (EDS) (INCA, Oxford Instruments) was carried out to determine the presence of a range of elements. For both dehydrated tissue sections containing adhesive gland cells and freeze-dried adhesive samples, EDS spectra were collected as area scans, point scans and elemental maps. In the case of point scans, it was possible to estimate weight % (mean ± internal error) of the elements present to quantify them, whereas only presence/absence information was available for elemental mapping and area scans.

### 2.3. Raman spectroscopy

Freeze-dried *L. anatifera* adhesive was prepared from four different specimens and carefully placed on clean glass slides. Raman spectra were obtained at random positions on the sample material using a Horiba LabRAM II Raman spectrometer. The instrument is equipped with a 600 groove mm^−1^ diffraction grating, a confocal optical system, a Peltier-cooled CCD detector (255 × 1024 pixel array at −67°C) and an Olympus BX41 microscope arranged in 180° backscatter geometry. Analyses were performed using a 785 nm laser channelled through a Leica L100X/0.75 objective, providing a laser spot diameter of approximately 1.5 μm. Measurements were conducted for 30–45 s over the spectral range 400–3200 cm^−1^, while suitable signal-to-noise ratios were generated using a minimum of two accumulations per measurement. Instrument operating parameters, spectral acquisition settings and spectra manipulation (e.g. fluorescence reduction) were controlled using LabSpec v. 5.78.24 (Horiba Scientific). Calibration of the LabRam instrument was performed before each analytical session and routinely between individual analyses using the Raman peak of a crystalline silicon wafer (520.2 ± 0.5 cm^−1^; [33]). Uncertainty associated with the generation of Raman peak positions based on replicate analysis of the silicon standard is ±1.0 cm^−1^ (2*σ*; 0.2%).

In many cases, peaks in Raman spectra can be assigned with great confidence to specific vibrations and functional groups. However, the molecular complexity of biological materials can make this process difficult. There remains a scarcity of information in spectra databases for the Raman characteristics of specific proteins, while the assignment of Raman peaks to particular modes of molecular vibration is often approximate. Such uncertainty is partly influenced by experimental conditions (e.g. laser wavelength, spectral resolution, ambient temperature) or where spectral interference between neighbouring molecules masks one or more target peaks. Likewise, organic matter is known to generate spectral fluorescence during Raman analysis [34], thus the acquisition of usable Raman spectra from biological material remains a challenge (cf. [35]).

For this study, the Raman spectrum (400–3200 cm^−1^) and the peak maximum intensity values occurring within it were evaluated by reference to the proportion of amino acids in the bulk adhesive, the reference Raman spectra for individual amino acids [26,36] and published Raman spectra in the literature.

### 2.4. Attenuated total reflectance Fourier transform infrared spectroscopy

Attenuated total reflectance Fourier transform infrared (ATR/FTIR) spectroscopy was used to analyse freeze-dried *L. anatifera* adhesive from four different specimens. The samples were not subject to deuterium oxide (D_2_O) treatment as barnacle adhesive is highly insoluble and cannot be brought into solution without denaturants and reductants, which would have affected the FTIR spectra [37]. FTIR spectra were obtained using a Shimadzu FTIR-8300 at 4 cm^−1^ resolution between 600 and 4000 cm^−1^ (20 scans per sample). Background measurements were taken before each scan to create a baseline for the spectra and minimize drift.

## 3. Results and discussion

### 3.1. Scanning electron microscopy with energy dispersive spectrometry

**Figure 3.**
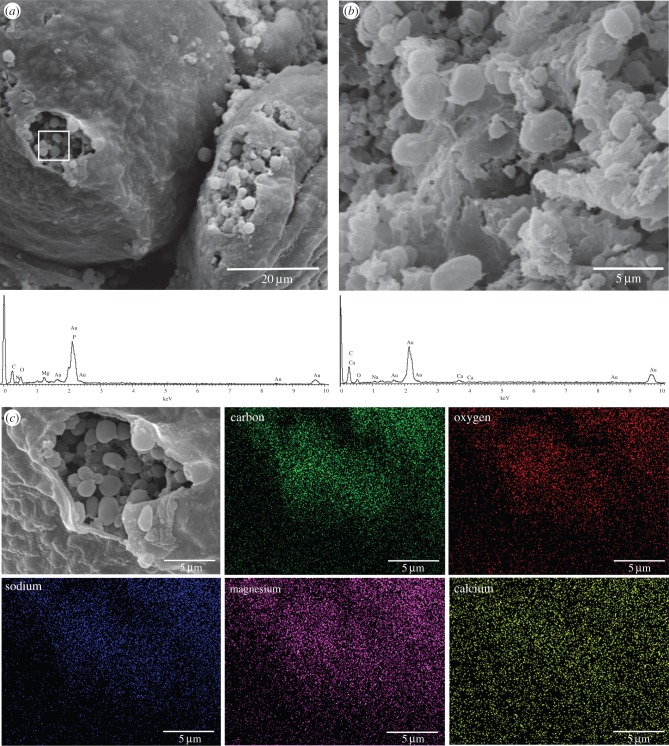
SEM-EDS of *L. anatifera* adhesive glands; (*a*) two adhesive glands with contents visible, callout box indicates location of area scan corresponding to EDS spectrum (below *a*). (*b*) High magnification of adhesive gland cell contents from a different specimen, asterisk indicates location of point scan corresponding to EDS spectrum (below *b*). (*c*) EDS maps (same specimen as *a*) showing spatial distribution of selected elements.

SEM showed that adhesive gland cells were large and located just inside the muscle layer at the periphery of the peduncle, amidst strands of connective tissue and adjacent to the ovarian follicles (figure 2). The surface of the adhesive glands was smooth; the contents of some adhesive glands were exposed, presumably due to rupture during sample processing. When this occurred, the ruptured glands contained many spherical globular components (figure 3*a*). Elements detected consistently in all adhesive gland samples were C, O, N (elements associated with the protein backbone), in addition to Na and Mg. In some samples Ca, Cl or P were also detected (figure 3*a–c*). In the tubeworm *Phragmatopoma californica*, distinctive granules containing Mg were present in the adhesive gland system [38], and theoretically could perform a cross-linking function (although this has not yet been confirmed experimentally). In the adhesive glands of *L. anatifera*, elemental mapping of C, O, Na and Mg appeared to reflect the structures observed in the sample (figure 3*c*); however P and Ca appeared as a random signal across the field of view. There was no apparent distinction between cell surface and cell contents; all detectable elements were present across the entire area examined, although this may have been due to the rupturing of cells during tissue processing.

**Figure 4.**
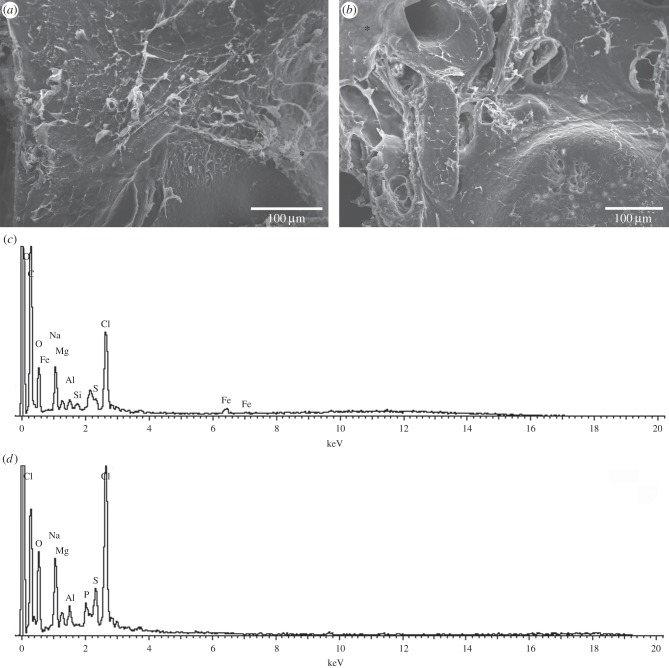
SEM micrographs of *L. anatifera* adhesive with EDS point analysis of locations marked with an asterisk. Spectra from point scans are shown underneath each SEM image: (*a*) surface of adhesive is primarily covered by a network of fibres or is a smooth surface with no fibres (as observed in bottom right corner of image), with (*c*) corresponding EDS spectrum; (*b*) surface of adhesive is interrupted by large pores (top left) and small pores (bottom right), with some fibrous structures and (*d*) corresponding EDS spectrum.

In comparison, the number of detected elements in the freeze-dried barnacle adhesive was far higher than what was observed in the glands (figure 4). The elemental constituents of the protein backbone (C, N, O), plus the additional elements commonly seen in the adhesive glands (Na, Mg, Cl) were detected in most of the samples. In addition, all adhesive samples contained S, which was not detected in the adhesive glands. Some adhesive samples also contained Ca, Al, Si, K and Fe; however, P was detected only once (from a total of 44 EDS scans—figure 4*d*). In previous elemental reports, Al and Si were concluded to have originated from the aluminium foil and PDMS substrates used during analyses [8,22]. As these substrates were not used in this study, this ‘contamination’ can be ruled out and we can therefore confirm the presence of Al and Si in barnacle adhesive. Presence of S and Ca in *L. anatifera* adhesive is in agreement with reports in other barnacle species [14,22]. Quantitatively, the weight percentage of specific elements was mostly consistent across scans (electronic supplementary material, table T1), with the elements Cl and Na having much higher abundances than the other elements. When present, Fe was moderately abundant. S was quite variable and though it was sometimes moderately abundant, this element was low in other scans. Thereafter, Mg, Al, P (where present) and Si (where present) were relatively low in abundance.

The increase in elements observed in the cured adhesive relative to the gland tissues may be explained by two alternatives: (i) in the gland, the adhesive precursor material is in a dilute form, and the concentration of some adhesive elements is too low to detect with EDS (the detection limit of this method is approx. 0.1%); alternatively (ii), differences could reflect chemical aspects of the respective environments of the samples. For example, Al and Si may originate in seawater, or in diatoms within seawater in the case of Si. In addition, a seawater signal may explain the ions Na^+^, Cl^−^, Mg^2+^, K^+^, SO_4_^−^ and Ca^2+^, which together make up more than 99% of the salts in seawater [39]. Indeed, a seawater origin may have accounted for variable detection of the elements Al, Si, Na, Cl, Mg, K and Fe in the cured adhesive in this study. These elements may be incorporated into marine adhesives in a similar way to their inclusion into fish otoliths [40] and variability in elemental signatures from adhesive samples may reflect the oceanic origin of the individual barnacles sampled.

A pertinent question is whether the elements that are incorporated into the adhesive have any functional role? For example, owing to its ability to form dimers by means of covalent bonds, there has been much interest in what form S may take in barnacle adhesive. Sulfur was detected by SEM-EDS in all cured adhesive samples from *L. anatifera* (although it was not detected in the adhesive glands). It has also been detected previously using comparative methodology (*Amphibalanus improvisus* adhesive [22] and *Dosima fascicularis* adhesive [14]). Sulfur-containing amino acids, Cys and Met, are generally present in various barnacle species, albeit in highly variable proportions (table 1); indeed, the abundance of both Cys and Met are very low in *L. anatifera* (table 1). Raman and ATR/FTIR analyses were used to shed light on what form S took inside the adhesive (see below).

**Table 1.** Adhesive amino acid compositions (residues per 1000) from membranous-based stalked barnacles *Lepas anatifera* [41] and *Dosima fascicularis* [42], and calcareous-based acorn barnacles *Megabalanus rosa* [12], *Balanus crenatus* and *Chirona hameri* [43].

AA	stalked/membranous barnacles		acorn/calcareous barnacles		
	*L. anatifera*	*D. fascicularis*	*M. rosa*	*B. crenatus*	*C. hameri*
Leu	97.9	97.6	82.8	81.0	87.8
Gly	95.7	87.1	79.2	85.9	82.7
Glu	91.0	114.1	91.5	86.3	90.5
Ala	89.9	98.8	74.7	64.7	68.7
Asp	87.5	101.2	90.7	82.8	78.9
Ser	87.5	98.8	99.1	76.9	113.6
Val	66.5	77.6	72.5	21.9	27.4
Arg	60.7	61.2	56.0	61.3	58.5
Ile	59.1	68.2	53.0	53.4	44.3
Thr	52.5	63.5	70.5	62.3	65.6
Pro	49.9	43.5	49.2	60.6	83.9
Lys	38.5	24.7	56.7	67.9	54.7
Phe	36.9	49.4	37.1	39.8	36.7
Tyr	36.9	1.2	41.8	53.8	49.2
His	27.0	4.7	13.3	21.6	22.8
Cys	11.0^a^	2.4^a^	16.0^b^	72.8^a^	68.1^a^
Met	5.6	5.9	16.0	6.7	7.2

^a^Cystine/2 (dimeric state was measured).

^b^Cysteic acid.

### 3.2. Raman spectra of barnacle adhesive

**Figure 5.**
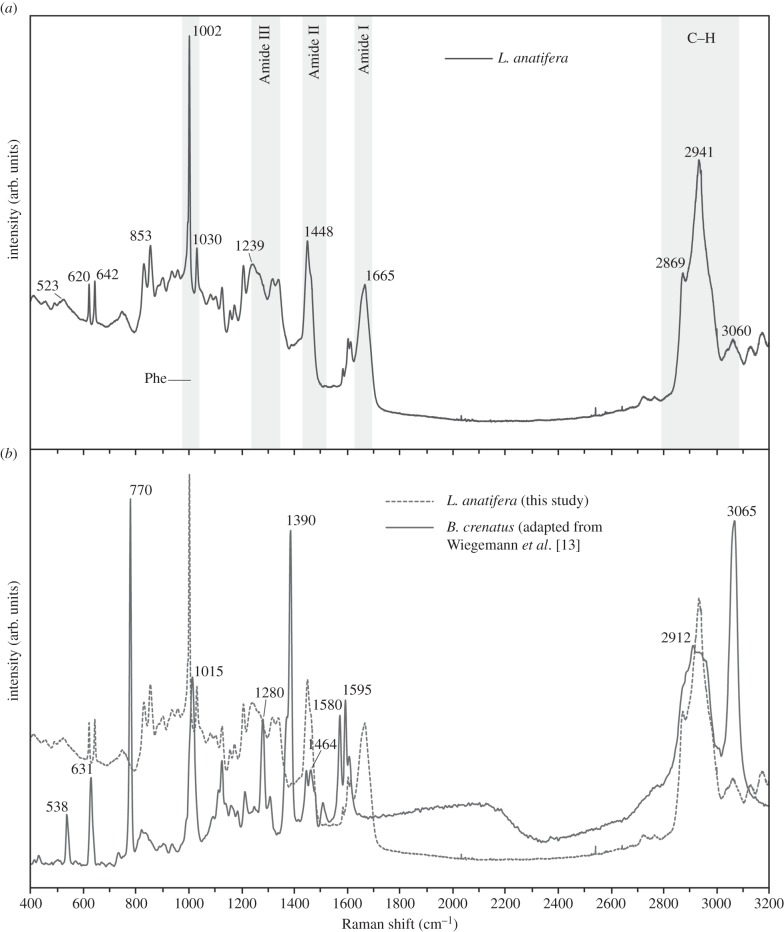
(*a*) Representative Raman spectrum from adhesive samples of *L. anatifera*, showing absence of Cys peaks (660–685, 500–540, 2500 cm^−1^); (*b*) Raman spectrum of the adhesive from *L. anatifera* (dashed line) compared to *B. crenatus* (continuous red line), with prominent peaks that are unique to each species labelled. The data for *B. crenatus* are reproduced with permission from Wiegemann *et al*. [13] and Springer Science & Business Media B.V. Springer 2005, arb. units: arbitrary units of intensity in Raman shift.

Four biological replicate samples of freeze-dried adhesive from different specimens of *L. anatifera* were subjected to Raman spectroscopic analysis and similar spectra were returned for all samples; for simplicity, one representative spectrum is shown (figure 5*a*). The strongest peak, at 1002 cm^−1^, is assigned to the phenylalanine (Phe) ring. Strong peaks also arose from a marked protein fingerprint, in the range of the Amide I band (primarily C=O bonds, at 1630–1680 cm^−1^) and the Amide II band (primarily N–H, some C–N, at 1440–1490 cm^−1^), with weaker peaks in the range of the Amide III band (mostly C–N, some N–H, at 1250–1350 cm^−1^). Peaks with a Raman shift from 2800 to 3100 were also very striking; these were due to C–H bonds arising from the protein backbone [44]. The broad, weak peaks at the low end of the scale may be due to small contributions from lipid (approx. 415 cm^−1^) [44].

The transition metals (chiefly Fe) play functional roles in other underwater adhesives, such as mussels [27] and gastropods [17]. Such protein–metal interactions have previously been shown to dominate Raman spectra, resulting in large peaks between 400 and 650 cm^−1^ in mussel byssus [27,45]. This study found no evidence of these large peaks in Raman spectra of *L. anatifera*, and there was no evidence that the Fe occasionally detected in cured *L. anatifera* adhesive during SEM-EDS plays a functional role.

To aid deeper interpretation of the Raman signal, the total amino acid content of the adhesive (table 1) and reference to the literature suggested the most probably assignments of peaks (table 2). Bands that indicate tyrosine (Tyr) were observed; a doublet at approximately 830/850 cm^−1^ is considered to be the primary marker of Tyr and the stronger 853 cm^−1^ peak observed in the current results could indicate that the Tyr is ‘exposed’ and available to act as both a hydrogen donor and acceptor [47]. Two common amino acids in barnacle adhesive, alanine (Ala) and serine (Ser), are also characterized by strong bands at 853 cm^−1^, and these probably contributed to the strength of the 853 cm^−1^ peak in the *L. anatifera* spectrum, particularly as *L. anatifera* adhesive contains a relatively low amount of Tyr (3.69%, [41]) (table 1).

**Table 2.** Amino acid (AA) assignments of the peaks in Raman spectra of barnacle adhesive which were based on peak maximum Raman shift value (cm^−1^ ± 5) and AAs ordered according to those which are most proportionally prevalent in *L. anatifera* adhesive. INT, intensity; br, broad; w, weak; m, moderate; o-p, out of plane; s, strong; sh, shoulder; vs, very strong.

shift	INT	AA	vibration assignment	references
409	w-br	Ala (m)		[26,36]
523	w-br	Phe (w)	ring/C–C=O deformation	[26,36]
		Cys (m)	S–S stretching	[29,36,44]
620	w-m	Glu (m) Phe (m)	C–C twisting	[36] [26,36,44]
642	w-m	Pro (m) Tyr (m) Ala (w)	C–C twisting	[26,36] [26,36,44] [26,36]
750	w-br	Trp (s)	ring breathing	[26,36,44]
828	w-m	Ile (m) Tyr (s)	ring breathing	[36] [26,36,44]
853	w-m	Leu (m) Ala(s) Ser (s) Val (s) Ile (m) Pro (sh) Phe (m) Tyr (s)	CH_3_ rocking C–N–C stretching C–C–O/C–C–N stretching ring breathing ring breathing	[46] [26,36] [26,36] [26,36] [36] [26,44] [26] [29,36]
899	w	Gly (s)	C–N–C stretching	[26,36]
935	w	Asp (s) Thr (m)	O–H o-p-vibration	[36] [36]
958	w	Met (w) Pro (w)		[36] [36]
1002	s	Phe (s)	ring breathing	[26,29,36]
1030	w-m	Leu (w) Ile (m) Pro (w) Phe (s)	C–N/C–C stretching ring breathing ring breathing	[46] [36] [26,36] [26,36]
1124	w-m	Glu (w) Asp(w) Ser (m) Val (m) Arg (m)	NH_3_^+^ wagging NH_3_^+^ rocking	[26] [36] [36] [26,36] [26]
1154	w	Gly (w) Phe (m) Tyr (w)	C–H/ring deformation	[26] [26,36] [36]
1170	w	Ile (w)		[36]
1204	w-m	Lys (m)		[36]
1239	m	Leu (w) Ala (w) Pro (m)	CH_2_ torsion CH_2_ wagging	[36] [26,36] [26,36,44]
1316	m	Leu (w) Glu (m) His (s)	C–H deformation NH_3_^+^ rocking	[36] [26,36] [26,36]
1338	w-m	Asp (s) Trp (s)	C–H bending C–H bending	[36] [26,27,36]
1448	m	Lys (m) Phe (w) Met (m)	CH_3_ deformation	[46] [26,36] [36]
1604	w	Ala (w) Phe (m)	C–O–O^−^ stretching C–C bending	[36] [26,44]
1613	w	Ser (w) Tyr (m) Trp (w)	C=C stretching C=C stretching	[26] [36,44] [26,36,44]

As mentioned above, the precise form S may take within barnacle adhesive is interesting due to the possibility for involvement of disulfide cross-links and their contribution to adhesion or cohesion of the adhesive. Most relevant to this question is the dimer cystine; the Raman signal from this is strong but variable, as the signal from different S–S conformers varies between 500 and 540 cm^−1^ (S–S) [36,44,48]. In *L. anatifera*, there was no evidence of a strong Raman peak in this region; instead, a weak and broad peak was observed at 523 cm^−1^. Although this region overlaps with certain conformers of S–S (gauche–gauche–gauche and gauche–gauche–trans) at 510 and 525 cm^−1^, respectively [49], the presence of disulfide is not supported by other evidence. For example, there is a lack of C–S signal at approximately 660–685 cm^−1^ which is also relevant to cystine. Also, the weak signal centred on 523 cm^−1^ overlaps with bonds from Glu residues and ring deformation of aromatic amino acids [36]; the latter residues are likely to be present and may be alternative sources of this weak signal. The reason for the absence of C–S bonds (660–685 cm^−1^), which we might expect due to presence of Cys and Met in the bulk adhesive of *L. anatifera* (table 1), may be attributed to the very small quantities of these residues in this species (table 1, [41]); thus, the signal from C–S may simply be swamped by other molecules. Another observation is a lack of the monomer cysteine in *L. anatifera*, which produces a strong Raman peak at 2500 cm^−1^ due to S–H bonds [44,48]. Cysteine was also apparently absent from the *L. anatifera* FTIR spectra (see next section).

The Raman spectrum from *L. anatifera* was compared with that of *B. crenatus* (data were replotted from [13]). The comparison is useful since *B. crenatus* possesses a calcareous baseplate which is glued to the substratum, rather than a membranous base, like *L. anatifera* (electronic supplementary material, figure S1). Overlaying the two species we can see that they had significantly different spectra and that many of the peaks present in *L. anatifera* were absent in *B. crenatus* and vice versa (figure 5*b* and table 3).

**Table 3.** Suggested explanation of species-specific differences in Raman spectra of adhesive from *L. anatifera* (present study) and *B. crenatus* [13]. AA assignments based on species-specific differences in adhesive AA composition (reported previously [41,43], table 1) and AA Raman spectra [26,36].

*L. anatifera* (membranous)	assignment	*B. crenatus* (calcareous)	assignment
642	Ala	770	Cys, Lys
853	Ala, Ser, Val	1280	Lys, Pro
1030	Leu, Ile	1390	Cys, Lys, Pro
1239	Leu, Ala	1475	Lys
1665	Amide I	1580	Phe

A distinctive feature of the *B. crenatus* adhesive Raman spectrum was the lack of the Amide I signal. This is explained by Amide I being occasionally weak in Raman spectra (A. Hartwig 2014, personal communication). Also the Tyr doublet was absent in *B. crenatus*, which was surprising, as the adhesive of this species has more abundant Tyr residues than *L. anatifera* (table 1; [41,43]). A peak at approximately 520 cm^−1^, which falls within the region indicative of S–S bonds [36], was stronger and more distinct in *B. crenatus* than in *L. anatifera*. But again, presence of disulfide is not particularly supported because only one conformer of S–S is indicated (trans–gauche–trans) and this seems unlikely [49]. More importantly, as with *L. anatifera*, the C–S bonds (approx. 670 cm^−1^) that are expected in cystine were not present in *B. crenatus* and the S–H bonds (approx. 2500 cm^−1^), indicative of the monomer were also absent. On the basis of these results, cystine has been discounted in *B. crenatus* adhesion [13], even though amino acid analysis of the bulk adhesive indicates high cystine content in this species (table 1, [43]). The strong peaks at 770 and 1390 cm^−1^ in the *B. crenatus* adhesive spectrum (table 3) may be associated with several amino acids, including Cys, Lys and Pro [36].

The role of Cys and its importance in barnacle adhesive has been much discussed. A pronounced molecular motif containing Cys in the form Cys-Xaa-Xaa-Xaa-Xaa-Xaa-Cys is conserved across multiple acorn barnacle species within cement protein (cp)-20k [50]. The only example, so far, of this protein being apparently absent was in a species with a membranous base (*Tetraclita japonica formosana*, [51]). Because the cp-20k protein is reported to be a calcite binding protein and important for cementing the adhesive to the barnacle's own baseplate [52], we can hypothesize that this protein is not required in membranous-based species. This case is supported by the observation that calcareous-based barnacles, including *B. crenatus*, generally possess more abundant Cys residues than membranous-based species (table 1). However, evidence for S–S bonds themselves in *B. crenatus* was contradictory with the techniques applied in this study. Future studies will determine whether the cp-20k protein is truly absent from *L. anatifera*. It may be noted also that the binding ability of the cp-20k protein is not universally thought to involve disulfide bridges; some evidence has been presented that disulfide bonds in barnacle adhesive impart shape upon individual proteins, rather than cross-linking ([9]; see also [6] in relation to cp-52k). It is possible that shape itself interacts with particular binding specificity. For example, in anti-coagulant proteins, folds produced due to disulfide bridges in proteins presented functional Ca binding sites at critical points along the molecule [53].

### 3.3. Attenuated total reflectance Fourier transform infrared spectroscopy

**Figure 6.**
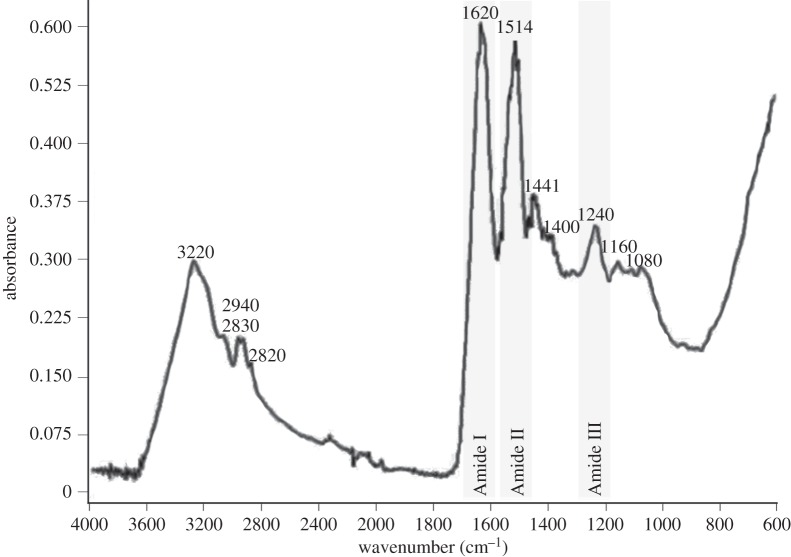
ATR/FTIR spectrum of *L. anatifera* adhesive, with prominent peaks labelled and the amide bands of proteins highlighted.

ATR/FTIR analyses of freeze-dried adhesive from four different specimens of *L. anatifera* showed highly consistent spectra (one is presented, for brevity, figure 6). No significant signature corresponding to peaks of C–S (680–820 cm^−1^) [54] or S–H (2550 cm^−1^) [55] were observed in ATR/FTIR, in line with the Raman data. The strongest peaks, at 1620 and 1514, were assigned to Amide I and Amide II, respectively, and a weaker peak at 1240 cm^−1^ corresponds to Amide III (1220–1350; [56]). The strong, broad peak at 3220 indicates Amide A and B, which result from N–H stretching of the protein backbone. As before, a comparison of FTIR spectra between species possessing different bases was possible; the species of comparison, with calcareous baseplates, were *Amphibalanus amphitrite* [57], *A. reticulatus* [7,54] and *A. improvisus* [22] (table 4).

**Table 4.** FTIR peaks (cm^−1^) for membranous-based stalked barnacle *L. anatifera* adhesive compared to calcareous-based acorn barnacles *A. amphitrite* [57], *A. reticulatus* [7] and *A. improvisus* adhesive. For *A. improvisus*, data are available on PMMA (left column) and PDMS (right column) [22]. Intensity: w, weak; m, moderate; s, strong; sh, shoulder. Peaks from spectra of dry or dry-pelleted adhesive, except where italics have been used.

assignment	*L. anatifera* (membranous)	Intensity	*A. amphitrite* (calcareous)	*A. reticulatus* (calcareous)	*A. improvisus*(calcareous)	
CaCO_3_				713	713	
CaCO_3_			872	876	876	
C–O stretching (carbohydrates)	1080–1160	w	*1081*	1055–1195	1090–1150	1120
Amide III	1240	m	1234			1250
Amide III			1313			
CaCO_3_, CH_3_ deformation	1400	w	1350–1420		1400	1390
CH_2_/CH_3_ deformation	1440	m	1425–1480	1428–1439		1450
Amide II	1514	s	1500–1580			1530
Amide I	1620	s	1600–1700	1625–1638		1650
C=O vibration				1793	1750	
S–H stretching				2516–2519		
C–H stretching	2820–2940	m	2800–3000	2835–3009		
Amide B	3200	sh	3060			
Amide A	3220	s	3280	3434

A calcium carbonate (CaCO_3_) signal indicated by prominent peaks below 1000 cm^−1^ was not present in *L. anatifera* adhesive, which was unlike all other species for which data are available (table 4). Carbonate also absorbs strongly at 1400 cm^−1^ [22] and *L. anatifera* shows a weak shoulder to be present in this region, but given the absence of the other relevant bonds, this was instead assigned to CH_3_ deformation [56]. This is significant because CaCO_3_ has been implicated in the adhesive process: barnacle adhesive was proposed to be predominately CaCO_3_ in a protein matrix [55], and this was supported by Berglin & Gatenholm [22], who concluded that CaCO_3_ is incorporated into the adhesive matrix during adhesive plaque formation. However, because the species examined previously all possessed calcareous baseplates, it was impossible in such cases to distinguish whether CaCO_3_ has been detected from the adhesive or from the baseplates (or, indeed, the lateral plates) [7,54,57]. In this study, ATR/FTIR analyses confirmed that the Ca content of *L. anatifera* adhesive (Ca was indicated by SEM-EDS scans) was not present in the form of CaCO_3_. We can conclude that the Ca signal in *L. anatifera* adhesive is not in the form CaCO_3_ and that the latter is not a prerequisite component of barnacle adhesion. Therefore, while micro-anchoring of calcium carbonate baseplates in an adhesive protein matrix [6] may apply to certain species, this cannot apply as an adhesive aid to *L. anatifera*.

There was no indication of phosphorylated proteins in the ATR/FTIR spectrum of *L. anatifera* (970 cm^−1^, [56]). Phosphorylated serine has been ruled out in the adult *L. anatifera* glands during a previous immunohistochemical study [5]. It could therefore be the case that occasional P within the adhesive during mapping (SEM-EDS, this study) is due to environmental origin and uptake of this element from seawater. Absence of significant levels of phosphorylation in the barnacle adhesive system is a major departure from virtually all wet-adhesion animal models described to date, including the mussel, tubeworm, sea-cucumber, caddisfly larvae and kelp spores [58].

Recent evidence suggests that non-DOPA phenolics may have some role in barnacle adhesive, as evidence for phenolic ring structures (including Tyr) was seen in FTIR spectra of *A. amphitrite* adhesive [57]. This study could neither confirm nor refute this hypothesis as, without D_2_O treatment, the Amide II peak overlapped and obscured any possible contribution from phenolic rings. There was evidence of a Tyr doublet in *L. anatifera* adhesive (Raman spectrum), although the significance of Tyr in barnacle adhesion is difficult to reconcile with the species-specific variations in Tyr content, including extremely low Tyr content in stalked barnacle species *D. fascicularis* (table 1) [12,41–43].

Finally, a broad region with at least three individual peaks between 1080 and 1160 cm^−1^ is likely to be associated with the C–OH bonds of oligosaccharide or polysaccharide carbohydrates [56,57]. The polysaccharide signal seen in *D. fascicularis* [14] is very similar to *L. anatifera* with respect to its strength, shape and position. Carbohydrate was previously detected during histological studies of *L. anatifera* gland tissues [5] and may be linked with limited glycosylation previously reported in barnacles [6,59] or other polysaccharide sources, potentially including chitin; but this requires further studies.

## 4. Conclusion

(1) This is the first chemical analysis of the adhesive of *L. anatifera* using EDS, Raman spectroscopy and ATR/FTIR spectroscopy. The Raman analysis in this study (table 2) adds to a limited number of previous spectra from barnacle cement in cypris larvae [30], adult barnacle (acorn species) [13] and adult barnacle (stalked species) [14].
(2) A large variety of elements were observed in the adhesive, including C, N, O, Na, Mg, Ca, Cl, S, Al, Si, K and Fe; although P was detected only once in 44 scans. There was no evidence of Fe–protein complexes in *L. anatifera* adhesive during Raman, confirming previous reports and demonstrating a major point of contrast with mussel adhesives.
(3) The presence of P was mostly absent in *L. anatifera* adhesive and there was no evidence of phosphorylated proteins during spectroscopy. Involvement of phosphorylated serines is well known in other wet-adhesion models, including mussel, tubeworm, sea-cucumber, caddisfly larvae and kelp spores.
(4) While S and Ca were present in the adhesive of *L. anatifera*, there was little evidence to suggest that either of these elements play functional roles; evidence for presence of S–S, for example, was weak and ambiguous. Instead these elements may be incorporated into the adhesive from marine salts.
(5) CaCO_3_ was absent from ATR/FTIR spectra of *L. anatifera* adhesive; this contrasts with reports from other barnacle species and can be attributed to respective differences in the barnacle base that is adhered to the substratum. *Lepas anatifera* is adhered to the substratum via a membranous base, whereas the other species examined previously possess calcareous baseplates. As it is not universally present, we can conclude that CaCO_3_ is not a prerequisite component of barnacle adhesion in general.
(6) Significant differences were observed between the Raman spectra of *L. anatifera* and *B. crenatus*, which has a calcareous baseplate; the explanation for these spectral differences awaits further experimental work.
